# COVID-19: Regional Differences in Austria

**DOI:** 10.3390/ijerph19031644

**Published:** 2022-01-31

**Authors:** Hanns Moshammer, Michael Poteser, Lisbeth Weitensfelder

**Affiliations:** 1 Department of Environmental Health, Center for Public Health, Medical University Vienna, 1090 Vienna, Austria; hanns.moshammer@meduniwien.ac.at (H.M.); michael.poteser@meduniwien.ac.at (M.P.); 2 Department of Hygiene, Medical University of Karakalpakstan, Nukus 230100, Uzbekistan

**Keywords:** COVID-19, regional differences, ecological risk factors, underreporting, infection risk

## Abstract

In the turbulent year 2020, overshadowed by the global COVID-19 pandemic, Austria experienced multiple waves of increased case incidence. While governmental measures to curb the numbers were based on current knowledge of infection risk factors, a retrospective analysis of incidence and lethality at the district level revealed correlations of relative infection risk with socioeconomic, geographical, and behavioral population parameters. We identified unexpected correlations between political orientation and smoking behavior and COVID-19 infection risk and/or mortality. For example, a decrease in daily smokers by 2.3 percentage points would be associated with an increase in cumulative incidence by 10% in the adjusted model, and an increase in voters of the right-wing populist party by 1.6 percentage points with an increase in cumulative mortality by 10%. While these parameters are apparently only single elements of complex causal chains that finally lead to individual susceptibility and vulnerability levels, our findings might have identified ecological parameters that can be utilized to develop fine-tuned communications and measures in upcoming challenges of this and other pandemics.

## 1. Introduction

The COVID-19 pandemic caused by the SARS-CoV-2 coronavirus is in many ways unique in human history. It is the first time that a mainly aerial transmitted viral infection has hit the entire world in the situation of highly progressed general globalization, with severe implications that are not limited to direct health effects. In parallel, science has been confronted with the unprecedented situation of an enormous total amount of data [1,2] that allows for insights into mechanisms and interplay of the viral disease, e.g., [3] with specific aspects of human behavior. The global spread of the COVID-19 pandemic has already provided valuable information on the effectiveness of different risk minimizing measures that are of relevance for viral infections in general. Several sociodemographic risk factors for becoming infected have soon been investigated, such as an increased risk for becoming infected in the first wave for age groups of 65 years and above [4]. The interplay of social factors with viral transmission and related mortality includes causal interactions of disease and behaviors that are expected and easy to comprehend, such as the effect of social distancing or habits affecting personal hygiene and environments [5,6]. However, the large number of affected populations, as well as the cultural, geographic and social differences among them, now allow for the discovery of more subtle and sometimes even unexpected social factors affecting infection risk and/or lethality [7,8]. We here set out to investigate different personal habits and sociopolitical factors and their implications on COVID-19 risks on a district level in Austria. While the chain of causal elements from each observed factor to the related disease outcome is not resolved yet in most cases, similar observations have been made globally and may finally lead to a better understanding of the social aspects of infectious diseases in modern societies of the 21st century.

**Figure 1:**
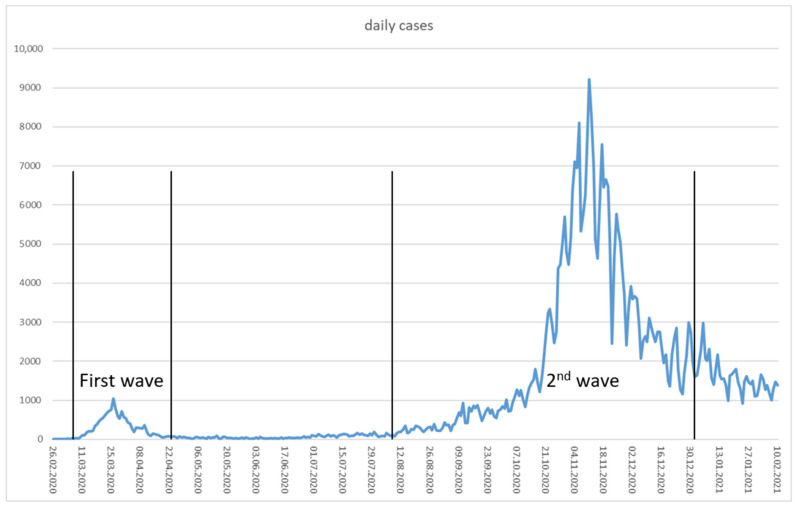
Time course of daily COVID-19 diagnoses in Austria.

In Northern Italy, SARS-CoV-2 caused a severe outbreak in February and March 2020. Many deaths and an overwhelmed healthcare system shocked health experts and the public all over Europe. Most likely introduced by Italian tourists, the virus also spread in Austrian skiing resorts that then served as an additional hub for the European spread [9,10]. While Austrian authorities first acted slowly in recognizing the danger, quarantining the tourist village of Ischgl was imposed as one of the first actions. The announcement of quarantining the region on 13 March made tourists flee the skiing resort back to their home countries without any proper control by health authorities [11]. Soon afterwards, on 16 March 2020, the Austrian Federal Government ordered a strict lockdown for the whole country, including closures of schools and shops, as well as cancelling of cultural and sports events. In the beginning of this first lockdown, there was a solid support for strong measures among the Austrian population. Some people were even afraid of helping others in need or of obtaining help themselves (own observation). In medical contexts, telemedicine as one method to reduce face-to-face-encounters became increasingly relevant [12]. Thus, the first wave of the COVID-19 epidemic in Austria was soon curbed successfully [10]. Figure 1 shows the daily diagnosed cases of COVID-19, as reported on the website of the Austrian Health and Food Safety Agency [13].

Even after the restrictions were relaxed in April, the number of new infections remained low throughout the summer in Austria, though some minor local outbreaks occurred in tourist places and in enterprises, usually with poor working conditions such as those in logistics centers and in slaughterhouses [14,15]. In spite of strict control measures, the virus also spread in several elderly care homes [16], causing a number of deaths that then triggered a variety of medial responses, raising the criticism of negligence of the disease in politics and society.

The government was reluctant to announce another lockdown in fall when case numbers were rising again. When the healthcare sector sounded the alarm because of overcrowded hospitals, new restrictions were announced, but restriction endorsement in the population was much weaker than in the first lockdown [17], and restrictions were seen as the biggest current concern in the Austrian population [18]. Nevertheless, the second, much more severe wave of COVID-19 incidence was also extenuated until before Christmas, but then stabilized at a relatively high weekly rate.

Indeed, the temporal course of case numbers, as depicted in Figure 1, is only one part of the story. The estimated number of undetected cases was especially high at the beginning of the epidemic, when diagnostic procedures were still lacking and testing facilities were still underdeveloped. In fall, PCR testing was broadly implemented with the capacities substantially increased. In addition, several antigen tests were then marketed and soon applied in large screening campaigns. Thus, it is difficult to compare later case numbers to those recorded during the first wave, amongst others, due to underdetection: Based on antibody screening tests, it was estimated that only one third or less of all first-phase cases were detected [19,20,21,22]. The detection rate was considerably better during the second wave in fall 2020 [23].

At the end of January 2021, new variants of the coronavirus started to spread in several parts of Austria [24,25]. National and local politicians, including the local communities and at the federal country level, tried to deflect responsibilities. Likewise, tensions increased between advocates of social distancing measures versus those of particular economic interests, between urban and rural areas, and between political parties.

As this pandemic qualifies as a crisis severely affecting health, economy, and the quality of life of the entire population, it was quite expected that the governmental handling of the epidemic would be of high political relevance. It is thus not surprising that unpopular measures introduced by the government have been used as arguments by opposing parties to express criticism in an emotionally heated general mood. Measures affecting social distancing, such as strict lockdowns with limited personal freedom or the prohibition of public gatherings were especially criticized as a sign of politically motivated suppression. Accordingly, a study published in the aftermath also found connections between voting behavior and COVID-19 vaccine hesitancy [26]. Tendencies of existing partisan differences regarding adherence to governmental measures were, in Austria, such as in other countries [27], additionally fostered by intentional misinformation and unsubstantiated conspiracy theories. While the political implications are still a matter of discussion, there is no doubt that politically motivated noncompliance to implemented governmental measures and the denial of scientific evidence regarding other protective behaviors may imply a real and measurable elevated disease risk within specific political groups. Without delving deep into the political preferences, it is evident that populist parties emphasizing the preference of personal freedom over social solidarity were more likely to exploit the inconveniences, restrictions, and shortcomings linked to governmental management to communicate and propagate their overall political agenda. In Austria, these criteria are met by the right-wing populist party (FP). 

Regional outbreaks vary regarding incidence between geographical regions and regarding percentage of severe cases. Different regional incidence rates might be caused by varying exposure of groups related to demographic backgrounds such as age or amount of working people, whereas at the first glance, not obviously related factors might also have an additional influence. For example, voting behavior could be directly and indirectly linked to incidence rate, as in Austria, representatives of the right-wing populist party (FP) continued to propagate a notion that was contradictory to governmental measures and downplayed the danger of the disease. Accordingly, medial spread of unproven alternative theories (conspiracy theories) was shown to reduce public support of governmental regulations or physical distancing [28]. Hence, the preference or rejection of this political segment could also affect COVID-19 incidence rates. The voter base of political parties might differ regarding distribution of sex, age, or other sociodemographic factors. Generally, personal sociodemographic status has been shown to be related to compliance of preventive behaviors [29], with mothers being more conscious about preventions. 

Even smoking behavior might be connected both to incidence rate as well as severity of cases: Nonsmokers and less-frequent smokers have been shown to be more compliant to protective behavior in the early Japanese outbreak of COVID-19 [29], but smoking is also a risk factor for severe cases, being most likely associated with adverse outcomes [30,31], though there are some biologic mechanisms for both protective as well as detrimental effects of nicotine [32]. Furthermore, smoking behavior might also be connected to other risk-influencing factors: A previous study based on Austrian data [33] showed that smoking prevalence peaks between the ages of 25 and 34, and similarly a recent study from GB also showed a decline of smoking prevalence among older persons, starting already with an age over 25 years [34]. Recent data from Austria show that smoking behavior changed during the last decades, with a general decrease in daily smoking in men, while a decrease of daily smoking in women started only after 2014 [35]. According to these data, the percentage of never-smokers is now much more evenly distributed across age groups than in the previous study. 

Furthermore, sociodemographic and socioeconomic aspects might be of importance regarding case severity, such as general health status, that is demographically linked to age group and sex, but also socioeconomic factors such as income and educational level might be of importance: A US study showed that socioeconomic factors, especially educational level, play an important role in disease prevalence and mortality [36], and that low income was associated with more cases and fatalities [36]. Similar results can be found internationally: A Japanese study showed highest incidence and mortality in the group of the lowest income [37], a Swedish study showed that dying from COVID-19 mainly affects elderly, residents from nursing homes, and persons from less advantaged social groups [38], and an international review concluded that socioeconomically disadvantaged groups are hit harder by the pandemic [39]. Though sociodemographic or socioeconomic influences all point in the same direction, there still might be geographical differences regarding their effect: In a comparison of European countries, Austria ranked among the countries where sociodemographic influences on both case number and death number were highest, whereas the UK and Ireland ranked under countries with less impact of sociodemographic factors [40]. 

Aside from socioeconomic and sociodemographic factors, environmental and geographical factors such as sea-level might also be of importance for case rate and case severity: For very high altitudes of more than 2500 m above sea-level, altitude seemed to have a protective effect on case severity [41], and a Peruvian study concluded that for every 500 m increment in altitude, COVID-19 case rate was reduced by 22% and death rate by 40% [42].

Even air quality, which differs between geographical regions, also plays a role regarding associated amount of deaths [43,44,45,46], with poorer air quality being associated with more lethal cases and higher infection rates. Unfortunately, reliable information on representative air quality values per district was not available for the whole of Austria and the present study.

## 2. Materials and Methods

This investigation was designed as an ecological study, examining area-level parameters instead of individual factors. Generally, this approach bears the danger of ecological fallacy; that means misinterpreting area-level risks as individual risks. In addition to that possible fallacy, as in any observatory study, confounding is not prevented by design. Therefore, similar to in any other observational study and not restricted to ecological studies, confounding must be controlled for by including the possible confounders in the explanatory model.

Austria consists of nine federal countries. While most of the laws and, especially, health related laws in Austria are national, the control and implementation of these laws are usually in the hands of the federal countries that consist of districts as the core administrative units, at least as far as the healthcare sector is concerned. The federal countries of Austria and the number of districts in each federal country are provided in Table 1.

Daily COVID-19 case and death numbers per district were obtained from the COVID-19 dashboard of AGES (Austrian Agency for Health and Food Safety Ltd.: Vienna Austria). As can be seen in Table 1, the city of Vienna is one of the nine federal countries and houses nearly one quarter of the Austrian population. Vienna consists of 23 districts, but within-district differences in Vienna are often much more pronounced than between-district differences. In addition, mobility between districts in Vienna is very intense. Therefore, AGES decided to report COVID-19 cases for Vienna as a whole instead of reporting cases per district, but Vienna as a whole is huge compared to any other Austrian district. Therefore, including Vienna in an analysis of Austrian districts would massively distort the outcome, so we excluded Vienna from the district analysis, but provide a descriptive comparison of the federal countries (Table 1) including Vienna instead. 

### 2.1. Dependent Variables

We extracted the peak value and the cumulative number of COVID-19 cases per district as well as the cumulative number of COVID-19 deaths. We divided each of these numbers by the number of inhabitants of the district. These ratios were derived from count data that usually are not normally distributed. A visual inspection of residual plots confirmed that the residuals were not equally distributed. Therefore, these ratios were log-transformed, which provided a better fit of linear regression models. In addition to the case numbers per population, the deaths per diagnosed cases as a proxy of lethality were also calculated. This ratio did not need a log-transformation.

At first, all data from February 2020 until 10 February 2021 were examined. The relative risk mitigation performance of a district might have changed over the course of the epidemic. We therefore separated, somewhat arbitrarily (see Figure 1), the whole year into the first wave from 8 March until 23 April, the second wave from 9 August until 1 January, and the “endemic” period (between 24 April–8 August). We extracted the same dependent variables per district for each period as for the whole year, although “peak” values in the endemic phase that was indeed characterized by “no peak” are somewhat misleading.

### 2.2. Independent Variables

Characteristics of the districts were obtained from the Statistik Austria website. Population number per district (per 1 January 2020) was obtained from Statistik Austria [47] and percentages of inhabitants without Austrian citizenship and of inhabitants not born in Austria were obtained from Statistik Austria [48]. Most other parameters (2011 data) were obtained from communal statistics [49] where we extracted district data of total and habitable area, in hectares, to calculate population density per district, average size per household, number of working people, and number of people working in agriculture to calculate the percentage of the latter (“agriculture”), number of unemployed to calculate unemployment rate. We obtained 2019 data on percentage of the population aged less than 15 years and population aged 65 and older, and percentages in males from the Statistik Austria population report [50]. In addition, we extracted number of tourist-nights in 2019, which we divided by the population of the district (“tourism”), results of the last elections for parliament in 2019 (proportion of valid votes per all possible votes, proportion of votes for the five largest parties per all valid votes), and the political federal country. Altitude was defined as the altitude of the district capital and taken from an older study of melanoma risks [51]. Districts were either classified as urban or rural as follows: In Austria, large towns represent a district of their own (“urban”), while in most of the districts, a smaller town serves as the administrative center of a district that consists of that town and the surrounding rural area (“rural”). Smoking prevalence was obtained from the Austrian Health Information Survey (ATHIS) 2019 [52]. The ATHIS survey interviewed 15,461 Austrian subjects aged 15 or older. On a district level, that number was too small to provide robust estimates. Therefore, smoking prevalence (regular smoking, occasional smoking, no smoking) was reported on the level of the smallest administrative reporting unit of the healthcare system, usually consisting of 2–4 districts. We assumed the same smoking prevalence for each of the districts within one unit. In addition, we obtained district percentage of people 15 years and older with a completed secondary and a completed tertiary education, and the people working in another district as percentage of all working people from the register census 2011.

### 2.3. Statistical Analysis

Statistical analyses were performed in STATA Vers. 16.1. First, correlation coefficients were calculated between each dependent and each independent variable. Independent variables which were correlated as sufficiently strong (*p* < 0.1) with the outcome variables were considered for further examination in a multiple linear regression. Variables that were highly positively correlated with each other because they describe the same or similar concepts (e.g., percentage of foreigners described either by place of birth or by citizenship, R = 0.991) or are highly negatively correlated because they describe alternatively exclusive characteristics such as current smokers versus never-smokers (R = −0.884), high percentage of young versus elderly population (R = −0.841), or voters of different political parties (high absolute R-values especially between Social Democrats, Greens, and the right-wing populist party), could not be entered into the multiple linear regressions together because of collinearity. In the case of such high pairwise correlation, only the variable which displayed the stronger correlation with the dependent variable of interest was chosen as a predictor. Variables that did not contribute significantly to the outcome were omitted from the model by stepwise exclusion of the least significant variable. The final models included predictor variables with a *p* < 0.1, but only variables with a *p* < 0.05 were considered significant in the outcome. As the execution of health-related laws and regulations is in the hand of the federal countries, we next included the federal country as a nominal variable in that final model as a kind of sensitivity analysis. All analyses were performed with analytical weights for the population number per district.

## 3. Results

**Figure 2:**
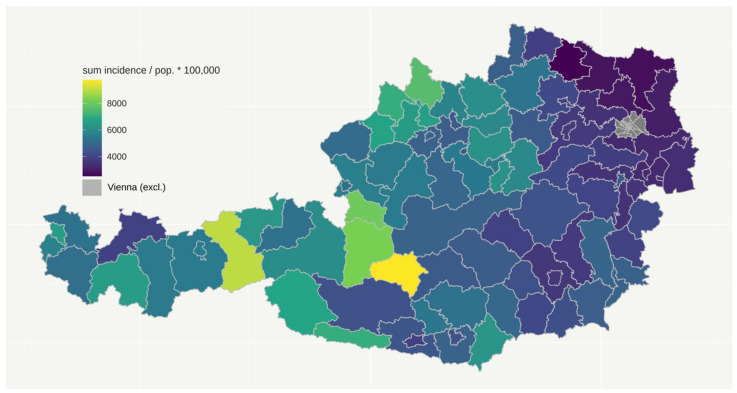
Incidence per population in Austrian districts until 10 February 2021.

**Figure 3:**
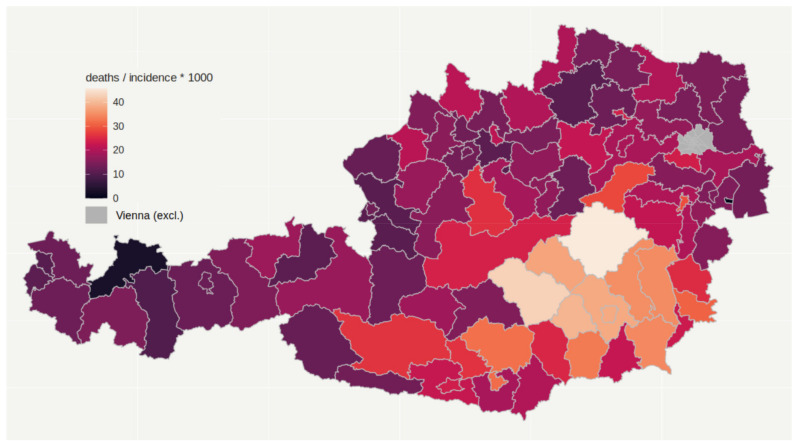
Deaths per incidence in Austrian districts until 10 February 2021.

We examined the parameters peak daily infections, total number of infections, total number of deaths (all per population number), and deaths per infection. A map of Austrian districts shows incidence per population (Figure 2) and deaths per incidence (Figure 3) during the period observed (February 2020 till February 2021). Peak daily infections were highly correlated to total number of infections, so results regarding peak values are not displayed, but are available upon request. The federal countries displayed some heterogeneity regarding the investigated parameters, but the ranking of the federal countries differed between the parameters.

Infection numbers and deaths in Vienna, the federal country not included in the detailed analysis, were well within the range of the per-capita counts of other countries. This was true for the whole period of investigation (Table 1), but also for the separate phases of the pandemic (data not shown). Ranking of the federal countries per parameter differed between the pandemic phases, and countries switched rank when controlling for additional factors, especially those that are not modifiable, such as altitude above sea level. In addition, some rural districts with smaller population numbers (around 10,000 or less) experienced no cases at all for a substantial duration of time but suddenly, maybe with the occurrence of a single cluster, suddenly ranked on top of all districts for a short period of time. This not only demonstrates that small numbers make poor statistics, but also that in rural areas people often felt that the pandemic is rather happening elsewhere and would not affect them directly. Thus, when the pandemic hit, they were less prepared, and the virus could spread more easily.

### 3.1. Cumulative Number of Daily COVID-19 Cases

**Figure 4:**
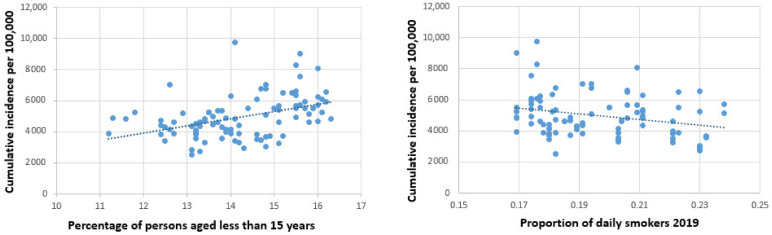
Scatterplots for young age (**left**) and daily smoking (**right**) with cumulative incidence.

The total number of COVID-19 cases amounted to (mean ± std. dev.) 4874.38 ± 1271.81 per 100,000. The respective number in the first wave was 181.79 ± 189.54, in the second wave 4485.28 ± 1213.8, and in the endemic phase between the two waves 52.42 ± 48.29. The final regression models for cumulative numbers are shown in Table 2, with and without consideration of federal country. Univariate correlation coefficients of all independent variables with the natural logarithms of cumulative case numbers can be found in Appendix A (Table A1). Higher altitude and higher percentage of young persons within the district population seemed to increase the risk consistently, though the increase became insignificant when controlled for federal country. Percent of daily smokers was associated with a reduced risk. For example, a decrease in daily smokers by 2.3 percentage points would be associated with an increase in cumulative incidence by 10% in the adjusted model (federal country not considered). More tourism only increased the risk in the first wave, while a larger number of household members seemed to be protective in the summer between the waves. Figure 4 illustrates the connection between age and smoking with cumulative case numbers in a scatterplot. 

### 3.2. Cumulative Number of COVID-19 Deaths (Mortality) and Lethality 

**Figure 5:**
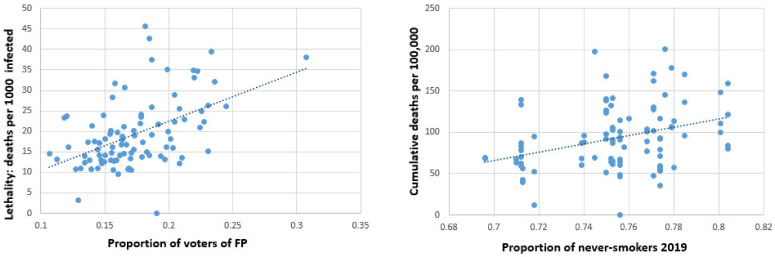
Scatterplots for right-wing voters and never-smokers on lethality and mortality.

The total number of COVID-19 deaths amounted to (mean ± std. dev.) 92.09 ± 36.88 per 100,000. The respective number in the first wave was 6.19 ± 6.95, in the second wave 81.85 ± 35.38, and in the endemic phase between the two waves 1.44 ± 1.84. The final regression models for mortality are shown in Table 3, with and without consideration of federal country. Univariate correlation coefficients for all independent variables with the natural logarithms of cumulative deaths can be found in Appendix A (Table A2). Both percentage of never-smokers and of voters of the right-wing populist party (FP) were consistently associated with higher risks of COVID-19 deaths. For example, in the adjusted model, an increase in voters of the right-wing populist party by 1.6 percentage points is associated with an increase in cumulative mortality by 10% (federal country not considered). Figure 5 illustrates the connection between right-wing voters (left) and never-smokers (right) with lethality (left) and mortality (right) in a scatterplot.

The final regression models for deaths per diagnosed cases as indicator of lethality are presented in Table 4. Univariate correlation coefficients between deaths per cases and independent variables are presented in Appendix A (Table A3). Overall, a higher percentage of inhabitants not born in Austria, as well as a larger average number of household members, tends to reduce the risk of dying from COVID-19, while the risk of dying was higher in a district with a high amount of FP voters (Table 4). 

Overall, our results show differences regarding several factors between the first wave and the rest, with tourist regions showing higher risks in the first wave.

## 4. Discussion

Some of our findings had to be anticipated, e.g., that case numbers were higher in areas with younger inhabitants, as we assumed that young people have a higher amount of social interactions [53,54]. However, this effect vanished when controlled for federal country. Regarding older ages, the percentage of inhabitants being 65 years and older remained a significant predictor, representing a protective factor on case numbers in the first wave and endemic phase. This means that—while age might remain a risk factor to the individual—according to our data, it is less risky to live in an area with a higher mean age. Nevertheless, as is well known from the literature [4,55], old age remained a risk factor for a severe course of disease and hence was positively correlated with mortality and lethality. 

An initial apparent connection between altitude and case numbers did not hold when controlled for federal country, but sea level and federal country are hard to disentangle, as some federal countries are located at higher altitude than others. We originally assumed more mountainous districts to have better air quality, and air pollution represents a risk factor [43,44,45,46]. A possible effect of sea level could also work via population density, since areas with lower population density in Austria are often at higher altitudes, and population density tended to seem protective—a finding we have already reported for the districts of Vienna [43]. Data from Tibet, Bolivia, and Ecuador [41] propose protective effects of altitude against COVID-19 infection, but the researchers’ findings relate to altitudes of more than 2500 m, where oxygen saturation of blood is already substantially lower. The habitable land of Austrian districts remains far below these extreme altitudes, and, for example, Pun et al. [56] do question a protective effect of altitude in general. In addition, our data do not confirm a possible protective effect of altitude. Similarly, more urban areas, which we expected to have poorer air quality, did not show a significant effect in the multivariate model, while they even tended to show lower infection rates in the endemic phase. 

In addition, contrary to our expectations, smoking prevalence is rather linked to a reduced COVID-19 infection risk. This effect in our data might be caused by several factors: Smoking behavior decreases with age and peaks in young adulthood [33,34,35], which is an age group that is generally at a lower risk level. We controlled for age in our model, but our categorization of age groups was not very detailed: In our data, young and middle-aged adults share the same category, while smoking percentages might already vary between them [33,34,35]. Therefore, part of the protective effect in our data could in fact be age-induced, but other factors might contribute to the seemingly protective effect of smoking as well: Even in a district with a higher percentage of smokers, nonsmokers are the majority, often disliking passive smoke. Hence, rooms where smokers stay are often better ventilated for the benefit both of smokers and nonsmokers. In addition, smoking in public restaurants or bars is not allowed in Austria, so smokers rather chat together outside in the fresh air, where transmission rates are lower. Finally, and in our opinion most importantly, this paper is about number of diagnosed COVID-19 cases only, not about the “true” number, which includes unreported cases. Especially with asymptomatic or mild cases, underdiagnosis was likely substantial. This was even more of an issue during the first wave, when seemingly protective smoking effects were most pronounced. Could it be that smokers underreport mild respiratory symptoms because they experience some respiratory symptoms on a routine basis anyways? Using a smartphone app, a British study showed that smokers reported more symptoms suggesting a COVID-19 diagnosis than nonsmokers [57]. In addition, among those who tested positive, smokers had a higher symptom burden and were more likely to need hospital care, but smoking rates were overall slightly lower in the group of those who tested positive. Smoking prevalence was generally lower in the tested group than in the study cohort [57]. Hence, the findings of the British study are compatible with our findings, while we assume underreporting in particular as an underlying reason for a seemingly protective effect of smoking on the infection rate. One should note that in our study daily smoking had a significant effect on case numbers and partly on number of deaths (mortality), but not on lethality. We also found living in an area with a higher number of never-smokers to be a risk factor for increased mortality. This finding was to our surprise, but could partly be induced by other, underlying behavioral patterns than smoking, per se, which could spark future research: Maybe some life philosophies could, on the one hand, be connected to a healthy lifestyle, and, on the other hand, to behaviors that might still be connected to a higher vulnerability regarding severe courses of disease (e.g., alienation from academic medicine). However, mostly, a direct effect of smoking and nicotine still needs to be clarified: The role of smoking in case severity is not cleared yet. Meta-analyses have not been conclusive: Smoking has been shown to increase COVID-19 mortality [31], but it has also been shown that current smokers have a reduced risk of infection [58], while former smokers have an increased risk of hospitalization [58]. One meta-analysis even came to the conclusion that the rate of hospitalized smokers is surprisingly low, so that nicotine should be investigated as potential therapeutic option [59], a suggestion that could also be found elsewhere [60]. Given the diverse results of studies, a final valuation of the complex effects is still missing—also against the background of our own data, where living in areas with a high amount of daily smoking increased mortality risk in the summer between the pandemics, while living in communities with a high amount of never-smoking increased mortality risk in the second wave and in the total year as a whole. No significant effect of smoking was seen in the final models on lethality. 

Our study was sparked by political dispute and arguments between regions as to whom to blame. We therefore purposely also included parameters of political preferences as obtained from the last national elections. As explained in the introduction, we would have expected a higher risk in districts with a higher percentage of voters of the populist right party (FP). To our great surprise the contrary was true, as COVID-19 case numbers were indeed negatively correlated with the percentage of FP voters. We assume that conspiracy beliefs, which are related to less adherence to preventive behavior [61], especially contribute to the lower case numbers in the specific group, since regular screening tests might be seen as a form of preventive behavior. Overall, pandemic fatigue (a sort of distress resulting from the ongoing pandemic) has been linked to nonadherence of protective health behavior, and it has been shown that being worrisome or feeling the pandemic being close decreases pandemic fatigue [62]—following this idea, beliefs neglecting the disease would lead to higher pandemic fatigue and hence higher nonadherence. Additionally to fatigue and conspiracy beliefs, personality differences in the voter base might also contribute to the lower reported case numbers: It has been shown that personality might be relevant regarding health-behavior endorsement for COVID-19 [63], even if the contribution of personality to people’s pandemic response is only very small when confounders are controlled [64]. Contrary to the lower reported case numbers in our study, a higher percentage of FP voters was predictive of a higher COVID-19 mortality. A direct biological mechanism by which political opinions can affect disease severity appears to be unlikely. Different political parties might attract voters from different socioeconomic backgrounds; hence, a risk could be caused indirectly. We controlled for potential confounders such as education and age distribution, though similar to our results regarding smokers, categories were not very detailed, so a small effect of socioeconomic factors cannot be completely ruled out, but the adverse effect of FP voters was especially strong for lethality. Therefore, again, we want to propose differential underdiagnosis as the most likely explanation for the reduced case numbers: Citizens who mistrust the government and oppose governmental measures might even doubt the existence of a pandemic, and maybe are less prepared to participate in regular screening tests or to co-operate freely in contact tracing efforts. As Kittel [65] showed, FP voters in Austria underwent COVID-19 testing significantly less frequently between mid-February and mid-March. Such behavioral aspects would lead to a higher rate of underdiagnosis in these citizens, and again this would affect less severe cases more strongly. In that case, the mortality numbers might be more accurate and the higher mortality risk in districts with a higher percentage of FP voters would not indicate a higher lethality or a general tendency to more severe cases, but, rather, in truth a higher infection risk that is hidden by underreporting in the case of less severe cases. 

It should also be noted that voting behavior and smoking habits have been shown to be not completely independent. Far-right voters were demonstrated to be more likely to be smokers [66]. In addition, smokers more often tend to be nonvoters [67]. In summary, these findings may indicate a segment of the population that is not likely to accept political decisions and is thus less likely to adhere to imposed measurements as well as to follow governmental recommendations. Hence, it seems of importance to especially involve those groups that might experience more pandemic fatigue to increase acceptance via finding creative solutions to motivate their peers [68]. 

While voting for the populist right seems a risk for severe cases, a higher amount of people living in one household seems to be protective: Case numbers were significantly lower, though an effect on lethality did not remain significant after controlling for federal countries. One reason might be that social contacts have a positive impact on the immune system in general [69]. Nevertheless, more research about the relevance of household members for case numbers and severity should be encouraged.

Generally, it should be mentioned that total sizes of our differences found were often small compared to the variation over time. Thus, federal countries and districts that ranked high for certain COVID-19 parameters during one wave ranked low during the other or during the endemic phase. In addition, it must not be forgotten that our study is based on official regional numbers only, which does not allow to control for individual factors. If, for example, someone has a second home or a weekend house in a different federal country and becomes infected in a different region to the region where he registers as sick, we cannot derive that from our data. Similar to other studies, it is a general limitation where included variables had to be reduced to a single parameter, e.g., altitude per district was defined as the altitude of the district capital, and further detailed differences could not be considered. 

Differences between urban and rural districts were generally not very pronounced and did not hold in a multivariate model. Nevertheless, rural districts were quick to place the blame on the city dwellers, and vice versa. Federal countries blamed each other, and politicians from the national government did not hesitate to blame neighboring countries. Our data do not provide evidence for blaming single regions though. Mistrust and strife between regions reached another level when fighting started for the scarce resources of vaccines, for example when one district that struggled with the outbreak of a new virus variant received a larger proportion of vaccines than the others, but no vaccination campaign in a single country can ensure absolute safety. The pandemic will only be overcome when it is overcome globally.

## 5. Conclusions

In conclusion, we did find some significant correlations in case numbers, as well as in numbers of severe cases, to several factors, ranging from sociodemographic data (e.g., age, amount of household members) to voting behavior. Underreporting might account for some surprising results, such as a seemingly protective effect of smoking or voting for populist right parties on case numbers. Voting for the populist right is also connected to more severe cases, so reaching these voter groups regarding health-endorsing behavior seems important from a public health perspective. While some of our differences found shall enhance future research, the differences in our data were overall rather small. Differences in severity of the pandemic between regions do not seem to justify a different distribution of vaccines in Austria. Deriving from our data, competitive behavior between regions might be counterproductive, since resulting conflicts and inconsistencies might reduce prevention endorsement, increase underreporting, etc. 

Thus, our data demonstrated some regional variation in infection and mortality risk. We did observe some area-level predictors for that risk, but overall, regional variation was less extreme than some exaggerated statements have claimed, and differences and factors varied across the phases of the pandemic.

## Figures and Tables

**Table 1:** Federal countries in Austria.

Federal Country	Number of Districts	Number of Inhabitants	Peak per 100,000	Total Cases per 100,000	Deaths per 100,000
Burgenland	9	294,436	105.63	3846.00	74.04
Carinthia	10	561,293	150.72	4811.93	120.08
Lower Austria	24	1,684,287	67.09	3915.13	70.53
Upper Austria	18	1,490,279	151.31	5514.47	92.20
Salzburg	6	558,410	144.34	6399.24	84.35
Styria	13	1,246,395	87.77	4127.74	133.67
Tyrol	9	757,634	133.31	6019.40	79.33
Vorarlberg	4	397,139	202.20	5662.50	66.73
Vienna	23	1,911,191	101.61	4391.40	83.30
Austria (Total)	116	8,901,064	103.46	4782.11	90.43

**Table 2:** Coefficients of the final linear regression model for ln (1 + cumulative number per 100,000). Upper part: federal country not considered; lower part: federal country considered.

	Variable	Total Year	1st Wave	2nd Wave	Endemic Phase
Adjusted R^2^		0.435	0.464	0.199	0.196
	Sea level per 100 m	0.06553 **	−0.07372	0.06333 **	
	Percent below 15 years	0.10034 **		0.10133 **	
	Percent aged 65 and above		−0.12397 **		−0.16442 **
	Proportion vote for Soc. Dem.		−2.85619 *		
	Proportion valid vote	−0.90317 *		−1.03251 *	6.68207 **
	Tourism nights per population		0.00913 **		
	Proportion daily smoking	−4.10789 **	−8.47946 **	−4.51481 **	
	Av. Persons per household				−2.15088 **
Adjusted R^2^		0.696	0.529	0.662	0.625
	Sea level per 100 m	0.00026			
	Percent aged 65 and above		−0.14900 **		−0.14689 **
	Tourism nights per population		0.00807 **		
	Proportion daily smoking	−3.15999 **	−8.9770 **	−4.22585 **	
	Percent secondary education				0.039093
	Av. Persons per household	0.36510 **		0.35511 **	−2.58785 **

* *p* < 0.05; ** *p* < 0.005.

**Table 3:** Coefficients of the final linear regression model for ln (deaths + 1). Upper part: federal country not considered; lower part: federal country considered.

	Variable	Total Year	1st Wave	2nd Wave	Endemic Phase
Adjusted R^2^		0.267	0.106	0.308	0.040
	Sea level per 100 m	0.07152 **		0.0859 **	
	Percent aged 65 and above		−0.09137 *		
	Tertiary education	0.01934 *		0.01999 *	
	Proportion vote for Soc. Dem.			1.43921 *	
	Proportion vote for FP	5.83868 **		5.74163 **	
	Tourism nights per population		0.00683 *		
	Proportion daily smoking				7.09339 *
	Proportion never-smokers	5.01240 **		4.71345 **	
Adjusted R^2^		0.440	0.249	0.443	0.095
	Sea level per 100 m			0.00062	
	Percent aged 65 and above		−0.12746 *		−0.09126 *
	Proportion vote for FP	2.47332 *		3.52370 **	
	Tourism nights per population		0.00637 *		
	Proportion working in agriculture			−1.95950	
	Proportion never-smokers	5.13156 **		6.1099 **	

* *p* < 0.05; ** *p* < 0.005.

**Table 4:** Coefficients of the final linear regression model for death per 1000 cases. Upper part: federal country not considered; lower part: federal country considered.

	Variable	Total Year	1st Wave	2nd Wave	Endemic Phase
Adjusted R^2^		0.420	0.050	0.443	0.114
	Percent not born in Austria	−0.40551 *		−0.57471 **	
	Percent aged 65 and above	0.78620			
	Proportion vote for Conservatives	−32.15577 *		−26.15493 *	
	Proportion vote for FP	78.35056 **	185.3812 *	89.23756 **	
	Proportion working in agriculture			53.66657 *	624.5471 **
	Percent working	−0.70916 *			
	Av. persons per household			−35.2299 **	
Adjusted R^2^		0.701	0.233	0.676	0.226
	Percent not born in Austria	−0.34281		−0.36662	
	Percent aged 65 and above	1.13242 **		1.38660 **	
	Percent secondary education	−0.420333		−0.45343	
	Proportion vote for FP	58.4355 **	71.90519	57.37606 **	315.0977
	Proportion working in agriculture	−40.34525 *		−47.14581 *	309.686 *

* *p* < 0.05; ** *p* < 0.005.

## Data Availability

For the analysis, we used publicly available data only and we provide the sources in the methods section.

## Appendix A

In this appendix we present additional tables describing the results of the univariate regression analyses and our choice of variables for the multiple correlation.

**Table A1:** Correlation coefficients between cumulative numbers and the independent variable ln (1 + cumulative number per 100,000).

	Variable	Total Year	1st Wave	2nd Wave	Endemic Phase
Correlation coefficients					
	Percent no citizen	0.024	0.056	0.031	0.361 **
	Percent not born in Austria	−0.022	0.037	−0.015	0.400 **
	Urban vs. rural	−0.124	−0.052	−0.112	0.319 **
	Sea level	0.570 **	0.252 *	0.547 **	−0.330 **
	Percent male	0.33 **	0.258 *	0.314 **	−0.092
	Percent below 15 years	0.403 **	0.375 **	0.381 **	0.197
	Percent aged 65 and above	−0.322 **	−0.494 **	−0.293 **	−0.339 **
	Percent secondary education	0.020	−0.065	0.009	−0.400 **
	Percent tertiary education	−0.287 *	0.002	−0.268 *	0.298 **
	Percent working out of home	−0.057	0.019	−0.065	−0.126
	Proportion vote for Conservatives	0.282 *	0.358 **	0.224 *	−0.311 **
	Proportion vote for Soc. Dem.	−0.288 *	−0.503 **	−0.244 *	0.191
	Proportion vote for FP	−0.081	−0.266*	−0.058	−0.293 **
	Proportion vote for Greens	0.013	0.155	0.026	0.353 **
	Proportion vote for Liberals	0.030	0.204 *	0.032	0.166
	Proportion valid vote	−0.204 *	−0.045	−0.206 *	0.223 *
	Tourism nights per population	0.426 **	0.467 **	0.348 **	−0.197
	Proportion working in agriculture	0.148	0.063	0.121	−0.416 **
	Percent unemployed	−0.272 *	−0.080	−0.308 **	0.155
	Percent working	0.336 **	0.201	0.340 **	−0.046
	Population density	−0.144	−0.047	−0.132	0.386 **
	Proportion daily smoking	−0.379 **	−0.182	−0.385 **	0.128
	Proportion never-smokers	0.310 **	0.144	0.313 **	−0.096
	Av. persons per household	0.370 **	0.265*	0.339 **	−0.268 *

* *p* < 0.05; ** *p* < 0.005.

**Table A2:** Correlation coefficients between cumulative deaths and the independent variable ln (deaths + 1).

	Variable	Total Year	1st Wave	2nd Wave	Endemic Phase
Correlation coefficients					
	Percent no citizen	−0.24 *	−0.032	−0.209 *	0.005
	Percent not born in Austria	−0.277 *	−0.044	−0.241 *	−0.001
	Urban vs. rural	0.006	−0.070	0.023	0.102
	Sea level	0.252 *	0.059	0.223 *	−0.080
	Percent male	0.066	0.116	0.039	0.154
	Percent below 15 years	−0.179	0.146	−0.186	0.102
	Percent aged 65 and above	0.229 *	−0.245 *	0.247 *	−0.208 *
	Percent secondary education	0.164	0.066	0.130	−0.058
	Percent tertiary education	−0.208 *	0.099	−0.193	0.044
	Percent working out of home	−0.119	0.051	−0.120	−0.104
	Proportion vote for Conservatives	−0.069	0.176	−0.150	0.045
	Proportion vote for Soc. Dem.	0.175	−0.269 *	0.246 *	−0.124
	Proportion vote for FP	0.422 **	−0.073	0.417 **	0.036
	Proportion vote for Greens	−0.246 *	0.049	−0.218 *	0.037
	Proportion vote for Liberals	−0.357 **	0.044	−0.327 **	−0.045
	Proportion valid vote	−0.099	−0.006	−0.101	0.028
	Tourism nights per population	0.004	0.274 *	−0.072	0.086
	Proportion working in agriculture	0.281 *	0.054	0.222 *	0.101
	Percent unemployed	−0.030	−0.075	−0.044	0.089
	Percent working	0.007	0.120	−0.002	0.040
	Population density	−0.072	−0.038	−0.060	0.164
	Proportion daily smoking	−0.305 **	−0.100	−0.293 **	0.224 *
	Proportion never-smokers	0.364 **	0.044	0.350 **	−0.159
	Av. persons per household	0.063	0.107	0.027	0.029

* *p* < 0.05; ** *p* < 0.005.

**Table A3:** Correlation coefficients between deaths per cases and the independent variable death per 1000 cases.

	Variable	Total Year	1st Wave	2nd Wave	Endemic Phase
Correlation coefficients					
	Percent no citizen	−0.264 *	−0.115	−0.253 *	−0.205 *
	Percent not born in Austria	−0.275 *	−0.117	−0.262 *	−0.224 *
	Urban vs. rural	0.047	−0.036	0.053	−0.106
	Sea level	−0.025	−0.139	−0.021	0.052
	Percent male	−0.140	0.044	−0.167	0.244 *
	Percent below 15 years	−0.454 **	−0.116	−0.471 **	0.059
	Percent aged 65 and above	0.474 **	0.092	0.506 **	−0.065
	Percent secondary education	0.189	0.124	0.179	0.146
	Percent tertiary education	−0.074	0.113	−0.090	−0.157
	Percent working out of home	−0.069	0.054	−0.073	0.042
	Proportion vote for Conservatives	−0.225 *	−0.109	−0.259 *	0.237 *
	Proportion vote for Soc. Dem.	0.340 **	0.072	0.395 **	−0.243 *
	Proportion vote for FP	0.512 **	0.245 *	0.499 **	0.285 *
	Proportion vote for Greens	−0.298 **	−0.105	−0.298 **	−0.207 *
	Proportion vote for Liberals	−0.384 **	−0.143	−0.378 **	−0.183
	Proportion valid vote	−0.023	0.097	−0.036	0.060
	Tourism nights per population	−0.227 *	−0.128	−0.247 *	0.124
	Proportion working in agriculture	0.214 *	0.039	0.184	0.352 **
	Percent unemployed	0.093	−0.087	0.107	−0.097
	Percent working	−0.185	0.009	−0.207 *	0.178
	Population density	−0.023	−0.002	−0.03	−0.087
	Proportion daily smoking	−0.063	0.054	−0.072	0.086
	Proportion never-smokers	0.16	−0.041	0.171	−0.056
	Av. persons per household	−0.158	−0.063	−0.184	0.252 *

* *p* < 0.05; ** *p* < 0.005.
